# Can smoking history, peripheral inflammation, and nutritional status discriminate Parkinson’s disease? Development and validation of a clinically accessible nomogram

**DOI:** 10.3389/fnagi.2026.1848990

**Published:** 2026-07-23

**Authors:** Ke-ting Liu, Ze-min He, Li-hao Zhang, Li Xiao, Ling-qiao He, Yong-ming Wu

**Affiliations:** 1 Department of Neurology, Nanfang Hospital, Southern Medical University, Guangzhou, Guangdong, China; 2 Department of Neurology, The First School of Clinical Medicine, Southern Medical University, Guangzhou, Guangdong, China; 3 Department of Neurology, Chengdu Seventh People’s Hospital, Affiliated Cancer Hospital of Chengdu Medical College, Chengdu, Sichuan, China; 4 Department of Thoracic Surgery, The First People’s Hospital of Shuangliu District (West China Airport Hospital of Sichuan University), Chengdu, Sichuan, China; 5 Department of Human Anatomy, Development and Regeneration Key Lab of Sichuan Province, Chengdu Medical College, Chengdu, Sichuan, China; 6 Department of Information, Chengdu Seventh People’s Hospital (Affiliated Cancer Hospital of Chengdu Medical College), Chengdu, Sichuan, China

**Keywords:** discriminative model, nomogram, Parkinson’s disease, Prognostic Nutritional Index, Systemic Immune-Inflammation Index

## Abstract

**Background:**

Parkinson’s disease (PD) is a major public health challenge in China, with rising prevalence and limited early diagnostic tools. Systemic inflammation and nutritional status are increasingly recognized as key modulators of PD pathogenesis, but integrated predictive models remain lacking.

**Objective:**

To develop and internally validate a simple, clinically accessible nomogram for individualized probability estimation of currently having PD using routinely available demographic, lifestyle, and laboratory parameters.

**Methods:**

This retrospective study enrolled 2,221 participants (1,135 PD patients and 1,086 healthy controls) from a single center between December 2023 and December 2025. The cohort was randomly split into a training set (*n* = 1,555) and an internal validation set (*n* = 666). Least absolute shrinkage and selection operator (LASSO) regression was used for predictor selection from 25 candidate variables. Multivariate logistic regression was performed to identify independent associated factors and construct a nomogram. Model performance was assessed by discrimination (area under the receiver operating characteristic curve, AUC), calibration (calibration curve with Brier score), and clinical utility (decision curve analysis).

**Results:**

LASSO regression identified three core associated factors: smoking history, Systemic Immune-Inflammation Index (SII_100_), and Prognostic Nutritional Index (PNI). Multivariate logistic regression confirmed all three as independent associated factors: smoking history (OR = 0.66, 95% CI: 0.52–0.85, *p* = 0.001), SII_100_ (OR = 1.30, 95% CI: 1.25–1.36, *p* < 0.001), and PNI (OR = 0.96, 95% CI: 0.93–0.98, *p* < 0.001). The combined nomogram achieved an AUC of 0.802 (95% CI: 0.780–0.824) in the training set and 0.804 (95% CI, 0.771–0.838) in the validation set. Calibration curves showed acceptable but imperfect agreement (Brier scores: 0.185 in training, 0.188 in validation), with notable deviation in the training set but improved fit in the validation set, and decision curve analysis demonstrated positive net benefit across a wide range of threshold probabilities.

**Conclusion:**

The nomogram developed in this study may help identify individuals with higher probability of PD diagnosis in a cross-sectional setting using only three routine variables (smoking history, SII_100_, and PNI). The model maintained moderate discrimination (AUC ~ 0.80) and acceptable but imperfect calibration (Brier scores: 0.185 in training, 0.188 in validation), though calibration in the training set showed some deviation from the ideal line. Decision curve analysis supported its positive net benefit in real-world clinical decision-making. As a promising tool for cross-sectional diagnostic classification, it requires external validation to confirm its generalizability in large-scale populations.

## 1 Introduction

Parkinson’s disease (PD), as the second most prevalent neurodegenerative disorder, poses a significant threat to public health in China. Global Burden of Disease (GBD) data indicates that by 2021, the total number of PD patients in China had exceeded 5 million, with the country ranking first among G20 nations in both age-standardized incidence and prevalence rates (Xu et al., 2024; Su et al., 2025). Projections indicate that by 2050, the patient population in China will surge to 10.5 million (Su et al., 2025). This will position China as the country with the highest absolute number of PD patients and the highest age-standardized prevalence rate worldwide. This would make China the country with the highest absolute burden of PD worldwide. In light of this grim situation, it is crucial to develop effective tools for early detection and cross-sectional risk stratification.

The core pathological changes in PD involve progressive loss of dopaminergic neurons in the substantia nigra of the midbrain and abnormal aggregation of alpha-synuclein (Li et al., 2008; Luk et al., 2012; Kalia and Lang, 2015). By the time patients develop typical motor symptoms, dopaminergic neuron loss in the substantia nigra often exceeds 60% (Kalia and Lang, 2015), missing the optimal window for intervention. The disease may exhibit a prodromal phase lasting up to 20 years prior to the onset of motor symptoms (Kordower et al., 2008). During this stage, various non-motor symptoms emerge (Xu et al., 2024; Kalia and Lang, 2015), including hyposmia (reduced sense of smell), rapid eye movement sleep behavior disorder (RBD), and constipation. Although these early signals hold predictive value, they lack specificity. Furthermore, traditional PD diagnosis heavily relies on clinical symptoms, leading to high misdiagnosis rates and significant challenges in early recognition.

For a long time, research on PD has primarily focused on the loss of dopaminergic neurons and the aggregation of *α*-synuclein (Li et al., 2008; Ye et al., 2023). Recent evidence suggests that systemic and neuroinflammation are core mechanisms driving disease onset and progression, rather than merely “byproducts” of pathological changes (Ye et al., 2023; Lim et al., 2025). Persistent activation of the peripheral immune system can influence the central nervous system through multiple pathways, contributing to the entire pathological process of PD (Clarke et al., 2025; Greenland et al., 2025; Hoffman et al., 2025).

Regarding inflammatory markers, the Systemic Immune-Inflammation Index (SII_100_)—a composite indicator that integrates neutrophil, lymphocyte, and platelet counts—provides a more comprehensive reflection of systemic immune-inflammatory status and has been shown to be significantly associated with PD risk (Alagoz et al., 2025; Liu et al., 2025; Zhao et al., 2025; Zhou et al., 2025). At the same time, nutritional and immune statuses are closely intertwined. The Prognostic Nutritional Index (PNI), based on albumin and lymphocyte counts, is an important composite indicator for assessing nutritional-inflammatory status and has prognostic value in various diseases (Wang and Wang, 2019; Li et al., 2021; Zhang et al., 2023). However, its role in predicting PD risk remains to be explored. Smoking is one of the most consistent protective factors in the epidemiology of Parkinson’s disease (PD), but its value in comprehensive cross-sectional risk stratification has not yet been clearly defined (Wang et al., 2022; Rose et al., 2024a).

Currently, most studies examine the association between PD and only a single or a few biomarkers. Given that the pathogenesis of PD involves complex interactions among multiple pathways—including genetic, environmental, inflammatory, and metabolic factors—developing a predictive model that integrates multidimensional, readily accessible clinical and laboratory indicators may be more effective in identifying high-risk individuals than relying on a single indicator. Therefore, this study aims to develop and validate a risk chart model for personalized assessment of PD onset risk. Based on routinely collected demographic, clinical history, and hematological data, we will use machine learning algorithms (LASSO regression) to screen core variables from numerous potential associated factors, with the goal of providing a concise and practical clinical tool for cross-sectional case identification and diagnostic discrimination in this disease.

## 2 Methods

### 2.1 Study population

A retrospective collection of data was conducted for PD patients (inpatient or outpatient) at the Department of Neurology, Chengdu Seventh People’s Hospital, during the period from December 2023 to December 2025. Inclusion criteria were: (1) Diagnosis of according to the Chinese Diagnostic Criteria for PD (2016 Edition); (2) Han ethnicity, age ≥50 years. Exclusion criteria were: (1) Parkinsonian syndrome caused by vascular Parkinsonism or other diseases; (2) Patients with malignant tumors, autoimmune diseases, or hematological disorders; (3) Patients with chronic inflammatory conditions (including rheumatoid arthritis, vasculitis, inflammatory bowel disease, etc.); (4) Individuals with infection or fever within the past 3 months; (5) Use of antiplatelet agents (e.g., acetylsalicylic acid or clopidogrel) or nonsteroidal anti-inflammatory drugs; (6) Severe hepatic or renal insufficiency, unstable vital signs, or chronic liver/kidney disease. Control group inclusion criteria: Healthy Han Chinese adults aged ≥50 years with no known neurological, inflammatory, autoimmune, or malignant diseases.

**Figure 1:**
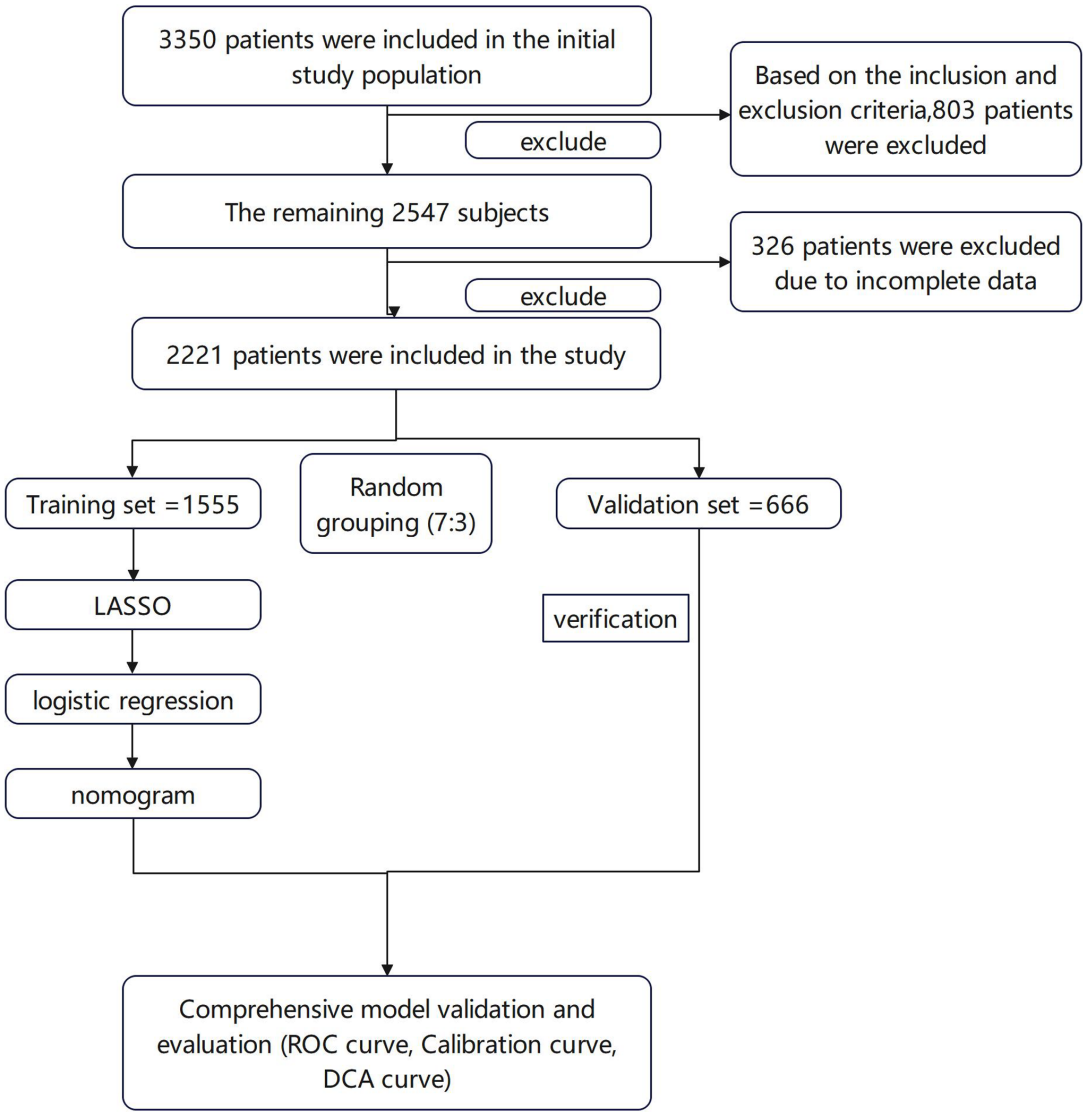
Flowchart of this study.

Exclusion criteria for the control group were identical to those for the PD group. This study was conducted in accordance with the STROBE guidelines (Cuschieri, 2019), and received approval from the Ethics Committee of Chengdu Seventh People’s Hospital. The research process strictly adhered to the Declaration of Helsinki (2013 revision) (World Medical Association, 2013). Sample size calculations were performed prior to study initiation (Latouche et al., 2004). The study flow diagram is presented in Figure 1. To assess the stability of the three-predictor logistic model, we performed 10-fold cross-validation on the entire cohort (*n* = 2,221). The results are described in the Results section and confirm the robustness of the model.

### 2.2 Data collection and potential predictive factors

Collect demographic data of patients (age, gender, body mass index [BMI]), lifestyle habits (smoking history, alcohol consumption history), past medical history (coronary heart disease, hyperlipidemia, diabetes, hypertension, chronic obstructive pulmonary disease [COPD]), and laboratory indicators. Laboratory indicators include: Systemic Immune-Inflammation Index (SII_100_), Prognostic Nutritional Index (PNI), White Blood Cell Count (WBC), C-Reactive Protein (CRP), fibrinogen, total protein, globulin, urea, creatinine (Cr), uric acid (UA), serum triglycerides, total cholesterol, low-density lipoprotein (LDL), and high-density lipoprotein (HDL). The calculation formulas for SII_100_ and PNI are: SII_100_ = (platelet count × neutrophil count/lymphocyte count)/100. As the original SII_100_ values have a large range, division by 100 brings the variable’s scale more in line with other indicators, which facilitates model fitting and interpretation of results. PNI = albumin (g/L) + 5 × lymphocyte count (×10^9^/L). Smoking history, alcohol consumption history, comorbidities, and other similar variables were recorded as binary variables (yes/no).

### 2.3 Dataset partition

The entire cohort of 2,221 patients was randomly divided into a training set (training cohort, *n* = 1,555) and an internal validation set (internal test cohort, *n* = 666) in an approximate 7:3 ratio. The training set was used for variable selection, model construction, and parameter estimation, while the validation set was used for the internal validation of model performance.

### 2.4 Predictor screening: LASSO regression

To identify core associated factors associated with Parkinson’s disease from the numerous variables, Least Absolute Shrinkage and Selection Operator (LASSO) regression was employed. Using Parkinson’s disease status as the dependent variable, all 25 clinical variables were included as candidate independent variables. A 10-fold cross-validation was used to select the penalty parameter *λ*. The final model was determined according to the “one standard error (1-SE) rule” (i.e., selecting the *λ* value corresponding to the most parsimonious model within one standard error of the minimum binomial deviance). Ultimately, variables with non-zero coefficients were retained as core associated factors.

### 2.5 Logistic regression analysis and nomogram construction

In the training set, the core predictive variables (smoking history, SII_100_, PNI) screened by LASSO regression were analyzed by univariate logistic regression, and the odds ratio (OR) and 95% confidence interval (95% CI) of each variable were calculated. In order to evaluate the collinearity between variables, the variance inflation factor (VIF) is calculated, and VIF < 5 represents the judgment standard of no serious collinearity. The three variables screened by LASSO regression were simultaneously included in the multivariate logistic regression model to analyze whether each variable was an independent predictor of Parkinson’s disease. Based on the results of the multivariate logistic regression, a nomogram for estimating the individual probability of currently having Parkinson’s disease (cross-sectional discrimination) was constructed. In the nomogram, each predictor is assigned a score based on its regression coefficient. The scores for all variables are summed to obtain a total score, which corresponds to a predicted probability of disease risk. This nomogram serves as a visual tool for individualized clinical risk assessment.

### 2.6 Model performance evaluation

#### 2.6.1 Discrimination

The receiver operating characteristic (ROC) curve was used to evaluate the discrimination ability of the model, and the area under the curve (AUC) and its 95% confidence interval were calculated. The AUC of the joint prediction model was calculated in the training set and the validation set respectively, and the AUC of each core variable single factor was calculated for comparison. The closer the AUC is to 1, the stronger the discrimination ability is.

#### 2.6.2 Calibration

The calibration curve is used to evaluate the consistency between the prediction probability and the actual observation probability of the model. Ideally, the calibration curve should coincide with the 45 ° ideal line. At the same time, Brier score is calculated as the quantitative index of probability prediction accuracy. The lower the Brier score is, the more accurate the prediction probability is.

#### 2.6.3 Classification performance

In the training set, set different risk score thresholds (≥0%, ≥30%, ≥60%, ≥90%, ≥100%), and calculate the sensitivity, specificity, positive predictive value (PPV), negative predictive value (NPV), accuracy, precision, recall and F1 score under each threshold. The optimal classification threshold is the threshold value when *F*1 score is the largest.

### 2.7 Clinical utility evaluation: decision curve analysis

Decision curve analysis (DCA) was used to evaluate the clinical net benefit of the model. DCA compared the net benefits of the prediction model with two extreme strategies (“intervene all patients” and “do not intervene any patients”) within a wide probability range of high-risk threshold. If the net benefit curve of the prediction model is higher than the two extreme strategies, it indicates that the model has clinical practicability.

### 2.8 Statistical analysis

This study used Zstats software and R software (version 4.4.0) with the rms, pROC, ggplot2, and dca packages for data processing and analysis. Continuous data following a normal distribution were presented as mean ± standard deviation (SD), while skewed data were presented as median (interquartile range, IQR). Categorical data were expressed as frequencies (percentages). All statistical tests were two-sided, and a *p*-value <0.05 was considered statistically significant. Binary logistic regression analysis was performed to identify independent associated factors (*p* < 0.05). A nomogram was constructed using the rms package in R. The discriminative performance of the model was evaluated using the area under the receiver operating characteristic curve (AUC), and calibration was assessed using calibration plots (calibration curves) with the Brier score.

## 3 Results

### 3.1 Study cohort and baseline characteristics

This study ultimately enrolled 2,221 eligible patients, including 1,135 patients with Parkinson’s disease (PD) and 1,086 controls. The entire cohort was randomly divided into a training set (*n* = 1,555) and a validation set (*n* = 666). There were statistically significant differences in the distribution of the Systemic Inflammation Index (SII_100_), Prognostic Nutrition Index (PNI), white blood cell count (WBC), C-reactive protein (CRP), and smoking status between the two groups (*p* < 0.001). Specifically, the SII_100,_ WBC, and CRP levels in the Parkinson’s group were significantly higher than those in the control group, while the PNI levels and smoking prevalence were significantly lower than those in the control group. Statistical analysis showed that there were no statistically significant differences between the training set and the validation set in terms of all the aforementioned clinical indicators, comorbidities, and Parkinson’s disease status (*p* > 0.05, Table 1). This suggests that the baseline characteristics of the two groups are well-aligned, the grouping is reasonable, and the reliability of subsequent model construction and validation results can be ensured. Detailed baseline characteristics of the patients are shown in Tables 1 and 2.

**Table 1:** Comparison of training and validation sets.

Variables	Total (*n* = 2,221)	Training set (*n* = 1,555)	Validation set (*n* = 666)	*p*
Age	71.69 ± 10.93	71.67 ± 10.94	71.74 ± 10.91	0.882
BMI	24.04 ± 3.24	24.05 ± 3.31	24.03 ± 3.06	0.930
SII	6.96 ± 6.60	6.86 ± 6.21	7.19 ± 7.42	0.270
PNI	47.20 ± 5.22	47.15 ± 5.15	47.32 ± 5.38	0.465
WBC	6.15 ± 2.13	6.12 ± 2.09	6.22 ± 2.23	0.319
CRP	5.87 ± 17.44	5.86 ± 17.14	5.87 ± 18.14	0.990
Fibrinogen	2.86 ± 0.70	2.85 ± 0.70	2.86 ± 0.69	0.776
Total protein	68.88 ± 6.19	68.97 ± 6.24	68.67 ± 6.06	0.299
Globulin	28.43 ± 4.59	28.53 ± 4.64	28.21 ± 4.47	0.123
Urea	6.28 ± 3.01	6.32 ± 3.17	6.17 ± 2.60	0.268
Cr	77.72 ± 45.48	77.56 ± 47.98	78.08 ± 39.06	0.807
UA	310.42 ± 92.03	310.45 ± 91.44	310.36 ± 93.46	0.983
Blood triglyceride	1.47 ± 0.98	1.46 ± 0.95	1.49 ± 1.05	0.524
Total cholesterol	4.32 ± 1.10	4.32 ± 1.10	4.31 ± 1.12	0.870
LDL	2.29 ± 0.81	2.29 ± 0.80	2.28 ± 0.83	0.780
HDL	1.46 ± 0.38	1.46 ± 0.39	1.46 ± 0.37	0.821
Parkinson’s disease				0.155
No	1,086 (48.90)	745 (47.91)	341 (51.20)	
Yes	1,135 (51.10)	810 (52.09)	325 (48.80)	
Sex 1				0.968
No	909 (40.93)	636 (40.90)	273 (40.99)	
Yes	1,312 (59.07)	919 (59.10)	393 (59.01)	
Coronary heart disease				0.752
No	2070 (93.20)	1,451 (93.31)	619 (92.94)	
Yes	151 (6.80)	104 (6.69)	47 (7.06)	
Hyperlipidemia				0.405
No	2031 (91.45)	1,427 (91.77)	604 (90.69)	
Yes	190 (8.55)	128 (8.23)	62 (9.31)	
History of diabetes				0.300
No	1929 (86.85)	1,343 (86.37)	586 (87.99)	
Yes	292 (13.15)	212 (13.63)	80 (12.01)	
History of hypertension				0.416
No	1,574 (70.87)	1,110 (71.38)	464 (69.67)	
Yes	647 (29.13)	445 (28.62)	202 (30.33)	
History of COPD				0.561
No	1971 (88.74)	1,376 (88.49)	595 (89.34)	
Yes	250 (11.26)	179 (11.51)	71 (10.66)	
Smoking history				0.763
No	1,534 (69.07)	1,071 (68.87)	463 (69.52)	
Yes	687 (30.93)	484 (31.13)	203 (30.48)	
Alcohol use history				0.607
No	1871 (84.24)	1,314 (84.50)	557 (83.63)	
Yes	350 (15.76)	241 (15.50)	109 (16.37)	

BMI, body mass index; SII, Systemic Immune-Inflammation Index; PNI, Prognostic Nutritional Index; WBC, white blood cell; CRP, C-reactive protein; Cr, creatinine; UA, uric acid; LDL, low-density lipoprotein; HDL, high-density lipoprotein; COPD, chronic obstructive pulmonary disease. For categorical variables, “yes” indicates the presence of the condition, and values in parentheses represent counts (percentages).

**Table 2:** Baseline characteristics of Parkinson’s disease group and non-Parkinson’s disease group.

Variables	Total (n = 2,221)	Non-Parkinson group (*n* = 1,086)	Parkinson’s group (*n* = 1,135)	*p*
Age	71.69 ± 10.93	71.81 ± 10.90	71.57 ± 10.95	0.599
BMI	24.04 ± 3.24	23.99 ± 3.03	24.09 ± 3.43	0.457
SII	6.96 ± 6.60	4.55 ± 3.68	9.27 ± 7.83	**<0.001**
PNI	47.20 ± 5.22	48.46 ± 4.73	46.00 ± 5.38	**<0.001**
WBC	6.15 ± 2.13	5.65 ± 1.72	6.63 ± 2.36	**<0.001**
CRP	5.87 ± 17.44	4.06 ± 13.05	7.60 ± 20.65	**<0.001**
Fibrinogen	2.86 ± 0.70	2.85 ± 0.68	2.86 ± 0.71	0.884
Total protein	68.88 ± 6.19	68.88 ± 6.15	68.87 ± 6.23	0.953
Globulin	28.43 ± 4.59	28.46 ± 4.57	28.41 ± 4.61	0.823
Urea	6.28 ± 3.01	6.30 ± 3.06	6.25 ± 2.96	0.653
Cr	77.72 ± 45.48	77.74 ± 46.28	77.69 ± 44.72	0.978
UA	310.42 ± 92.03	307.79 ± 89.80	312.94 ± 94.07	0.187
Blood triglyceride	1.47 ± 0.98	1.47 ± 0.90	1.48 ± 1.05	0.832
Total cholesterol	4.32 ± 1.10	4.33 ± 1.09	4.30 ± 1.12	0.648
LDL	2.29 ± 0.81	2.30 ± 0.81	2.27 ± 0.82	0.405
HDL	1.46 ± 0.38	1.46 ± 0.37	1.46 ± 0.39	0.759
Sex				0.363
Female	909 (40.93)	455 (41.90)	454 (40.00)	
Male	1,312 (59.07)	631 (58.10)	681 (60.00)	
Coronary heart disease				0.415
No	2070 (93.20)	1,017 (93.65)	1,053 (92.78)	
Yes	151 (6.80)	69 (6.35)	82 (7.22)	
Hyperlipidemia				0.868
No	2031 (91.45)	992 (91.34)	1,039 (91.54)	
Yes	190 (8.55)	94 (8.66)	96 (8.46)	
History of diabetes				0.686
No	1929 (86.85)	940 (86.56)	989 (87.14)	
Yes	292 (13.15)	146 (13.44)	146 (12.86)	
History of hypertension				0.878
No	1,574 (70.87)	768 (70.72)	806 (71.01)	
Yes	647 (29.13)	318 (29.28)	329 (28.99)	
History of COPD				0.075
No	1971 (88.74)	977 (89.96)	994 (87.58)	
Yes	250 (11.26)	109 (10.04)	141 (12.42)	
Smoking history				<0.001
No	1,534 (69.07)	660 (60.77)	874 (77.00)	
Yes	687 (30.93)	426 (39.23)	261 (23.00)	
Alcohol use history				0.828
No	1871 (84.24)	913 (84.07)	958 (84.41)	
Yes	350 (15.76)	173 (15.93)	177 (15.59)	

BMI, body mass index; SII, Systemic Immune-Inflammation Index; PNI, Prognostic Nutritional Index; WBC, white blood cell; CRP, C-reactive protein; Cr, creatinine; UA, uric acid; LDL, low-density lipoprotein; HDL, high-density lipoprotein; COPD, chronic obstructive pulmonary disease. For categorical variables, “yes” indicates the presence of the condition, and values in parentheses represent counts (percentages).

### 3.2 Screening of core predictive variables

**Figure 2:**
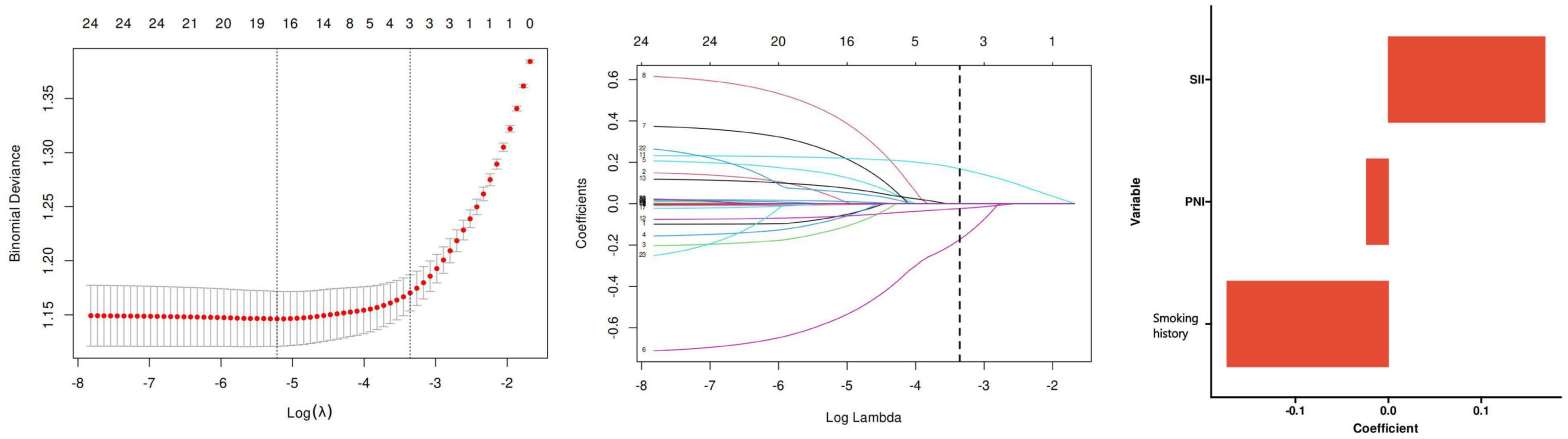
Column chart for research.

Based on the training set data, LASSO regression was used to select associated factors from 25 variables, and the regularization parameter *λ* was determined using the 1-SE criterion (*λ* = 0.0349073963559339). Ultimately, three variables with non-zero coefficients were retained: smoking history (coefficient = −0.42), SII_100_ (coefficient = 0.26), and PNI (coefficient = −0.04). The variable selection process is shown in Figure 2. These three variables were used for subsequent nomogram construction. To further assess the stability of the associations identified in the multivariate logistic regression, we performed 10-fold cross-validation on the entire cohort (*n* = 2,221) using the three core variables (smoking history, SII₁₀₀, and PNI). The analysis yielded a mean AUC of 0.853 (range 0.820–0.899), indicating consistent discriminative ability across folds. All three variables were selected in all 10 folds (100% selection frequency). The coefficients for smoking history ranged from −0.788 to −0.685, for SII₁₀₀ from 0.286 to 0.337, and for PNI remained stable at −0.006. Classification performance (at a threshold of 0.5) showed a mean accuracy of 0.790 (range 0.744–0.846), mean sensitivity of 0.810 (range 0.712–0.859), and mean specificity of 0.770 (range 0.718–0.835). These results confirm that the associations of smoking history, SII₁₀₀, and PNI with PD status are robust and not dependent on a specific data split.

### 3.3 Univariate and multivariate logistic regression analysis of core variables and construction of nomogram prediction model

**Figure 3:**
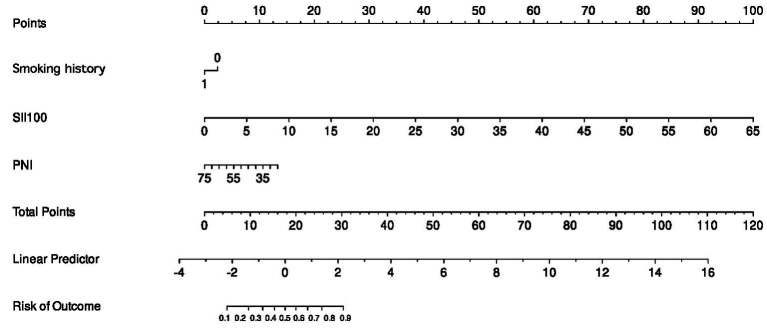
ROC curve of validation set.

Univariate logistic regression analysis showed that a history of smoking (OR = 0.46, 95% CI: 0.37–0.58), SII_100_ (OR = 1.35, 95% CI: 1.29–1.40), and PNI (OR = 0.91, 95% CI: 0.89–0.93) were all significantly associated with the onset of Parkinson’s disease (all *p* < 0.001). The variance inflation factors (VIF) for all variables were less than 2 (smoking: 1.021; SII_100_: 1.118; PNI: 1.101), indicating no multicollinearity. In the multivariate analysis, all three remained independent associated factors (history of smoking: OR = 0.66, 95% CI: 0.52–0.85, *p* = 0.001; SII_100_: OR = 1.30, 95% CI: 1.25–1.36, *p* < 0.001; PNI: OR = 0.96, 95% CI: 0.93–0.98, *p* < 0.001), with the direction of influence consistent with that observed in the univariate analysis (Table 3). These ORs reflect cross-sectional associations and do not imply causation, as noted earlier. Based on these three independent associated factors, a cross-sectional risk stratification nomogram for Parkinson’s disease was constructed (Figure 3). In this nomogram, smoking history is assigned a value of 0 (no history) or 1 (history), while SII_100_ and PNI are assigned scores corresponding to their actual measured values. The sum of the scores for each variable yields a total score, which corresponds to the probability of developing Parkinson’s disease. For a hypothetical subject with a smoking history (scoring 0 points), an SII100 of 8 (corresponding to approximately 14 points), and a PNI of 45 (corresponding to approximately 10 points), the total score would be 24 points. This corresponds to a Linear Predictor value of approximately 0.5 and a Risk of Outcome of approximately 0.65, indicating an estimated disease risk of about 65% for this individual. This provides a visual tool for rapid and intuitive clinical assessment.

**Table 3:** Results of single-factor and multi-factor logistic regression analysis of core predictive variables.

Variables	*N*	Event *N*	Univariate logistic regression		Multivariate logistic regression	
			OR (95% CI)	*p*	OR (95% CI)	*p*
Smoking history	484	189	0.46 (0.37–0.58)	<0.001	0.66 (0.52–0.85)	0.001
SII	1,555	810	1.35 (1.29–1.40)	<0.001	1.30 (1.25–1.36)	<0.001
PNI	1,555	810	0.91 (0.89–0.93)	<0.001	0.96 (0.93–0.98)	<0.001

### 3.4 Analysis of key variables and ROC curves for predictive models

**Figure 4:**
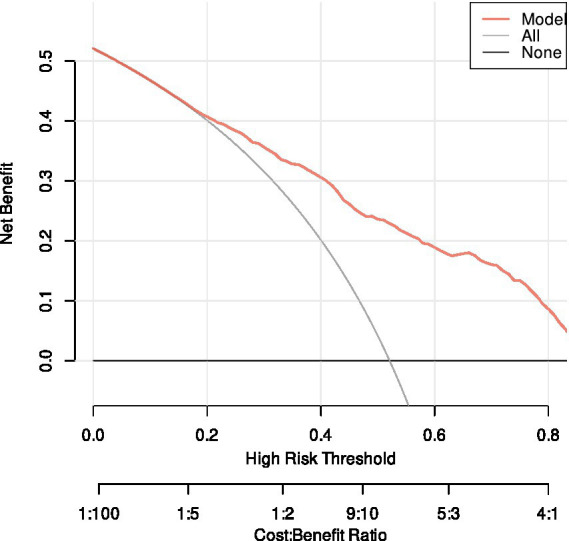
Decision curve analysis (DCA) of the nomogram in the training set. The *y*-axis represents the net benefit, a dimensionless ratio calculated as (true positives/*N*) − (false positives/*N*) × [threshold/(1 − threshold)]. The *x*-axis represents the high-risk threshold probability, i.e., the predicted probability above which an individual is classified as high-risk and considered for clinical intervention (e.g., specialist referral or confirmatory testing). The blue curve shows the net benefit of the nomogram across a range of threshold probabilities. The grey horizontal line (“none”) assumes no intervention, yielding zero net benefit. The black line (“all”) assumes intervention for every individual. The nomogram is considered clinically useful if its curve lies above both extreme strategies over a meaningful range of thresholds.

**Figure 5:**
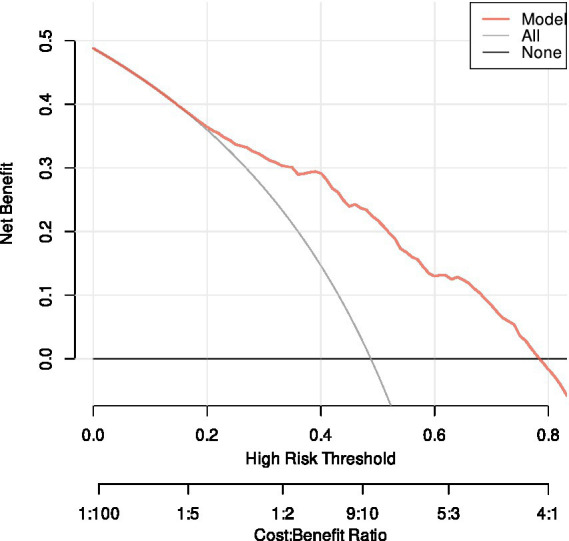
Decision curve analysis (DCA) of the nomogram in the internal validation set.

**Figure 6:**
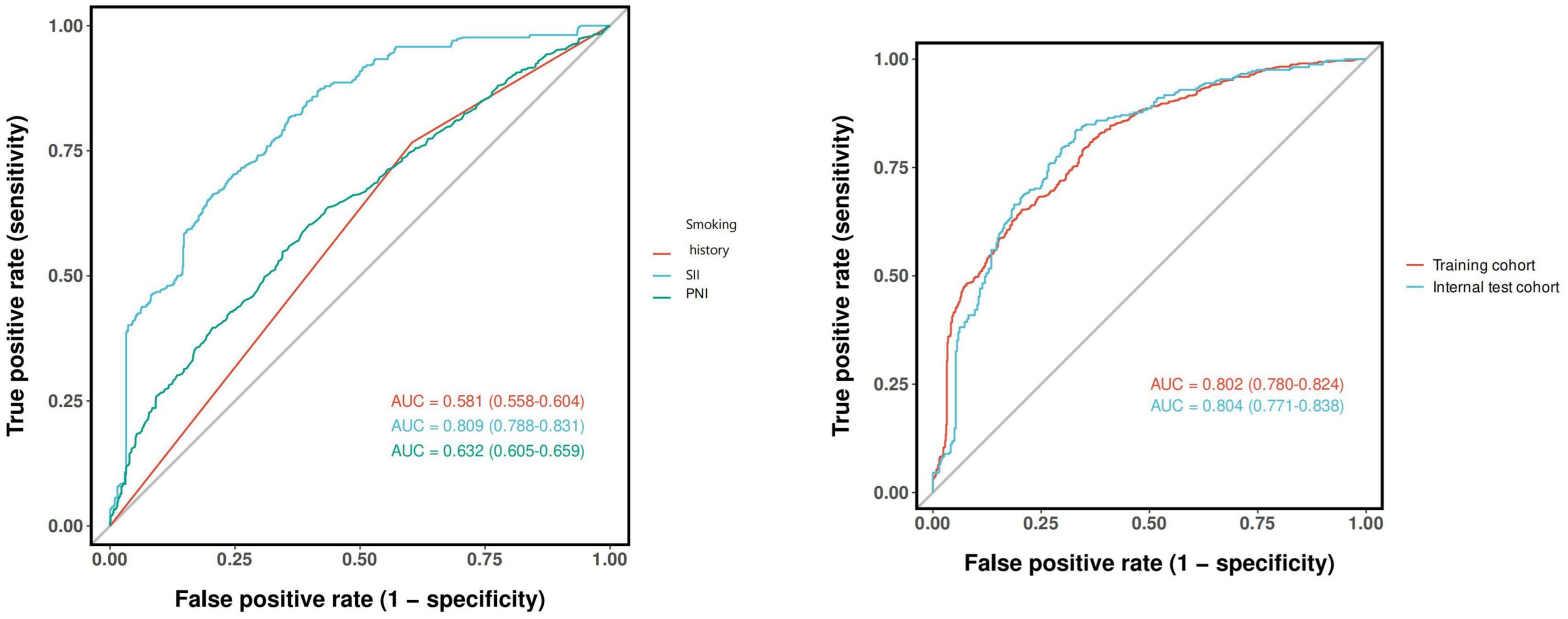
ROC curve of the training set.

Univariate ROC curve analysis based on the training set showed that SII_100_, PNI, and smoking history were all statistically significant associated factors of Parkinson’s disease (all *p* < 0.001). Among these, SII_100_ had the highest AUC (0.809, 95% CI: 0.788–0.831), PNI had an AUC of 0.632 (95% CI: 0.605–0.659), and smoking history had an AUC of 0.581 (95% CI: 0.558–0.604). The AUC for smoking history was below 0.6, suggesting that its discriminatory ability was poor when used alone. The combined predictive model constructed based on the above three variables had an AUC of 0.802 (95% CI, 0.780–0.824) in the training set and 0.804 (95% CI, 0.771–0.838) in the internal validation set. Regarding the observation that the combined nomogram achieved a slightly lower AUC (0.802) than SII~100~ alone (0.809), the 95% confidence intervals overlapped substantially (SII~100~: 0.788–0.831; combined: 0.780–0.824), indicating no statistically significant difference. Beyond AUC, the combined model offered better calibration (Brier score 0.185 in training, 0.188 in validation) and positive net benefit across a wide range of thresholds on decision curve analysis (Figures 4, 5). Moreover, incorporating smoking history and PNI enhances clinical interpretability and face validity. Therefore, while SII_100_ is a strong individual predictor, the combined nomogram is statistically non-inferior and provides a more balanced, clinically intuitive tool for cross-sectional risk stratification. The AUC of the combined model was highly consistent across the training and validation sets, indicating stable discriminatory ability and moderate generalization performance. The corresponding ROC curves are shown in Figure 6.

### 3.5 Classification performance of the predictive model at different risk thresholds

Table 4 shows the classification performance of the model in the training set at different risk score thresholds. As the risk score threshold increases from ≥0% to ≥100%, the model’s sensitivity gradually decreases from 100.0 to 0%, while specificity gradually increases from 0 to 100.0%, demonstrating a classic trade-off relationship between the two. The model’s accuracy ranged from 47.9 to 69.8%, the positive predictive value ranged from 52.1 to 83.9%, and the negative predictive value ranged from 47.9 to 85.4% across different thresholds. When the risk threshold was ≥30%, the model’s F1 score reached its highest value (0.740), with a sensitivity of 94.8%, a specificity of 33.0%, and an accuracy of 65.2%, representing optimal classification performance.

**Table 4:** Classification performance of the training set model across different risk thresholds.

Risk score threshold	Linear predictor cutoff point	Sensitivity (%)	Specificity (%)	PPV (%)	NPV (%)	Accuracy (%)	Precision (%)	Recall (%)	*F*1
≥0%	−Inf	100.0	0.0	52.1		52.1	52.1	100.0	0.685
≥30%	−0.8472979	94.8	33.0	60.6	85.4	65.2	60.6	94.8	0.740
≥60%	0.4054651	53.3	87.7	82.4	63.3	69.8	82.4	53.3	0.648
≥90%	2.1972246	14.8	96.9	83.9	51.1	54.1	83.9	14.8	0.252
≥100%	Inf	0.0	100.0		47.9	47.9		0.0	

The classification performance of the nomogram varied substantially across risk thresholds. The threshold that maximized the *F*1 score (a balance of precision and recall) was a predicted probability of ≥30%, which yielded a sensitivity of 94.8% and a specificity of 33.0%. While this threshold is useful for screening purposes—where missing a PD case is costly—the high false-positive rate (67%) makes it impractical as a standalone diagnostic rule.

### 3.6 Analysis of the calibration of predictive models

**Figure 7:**
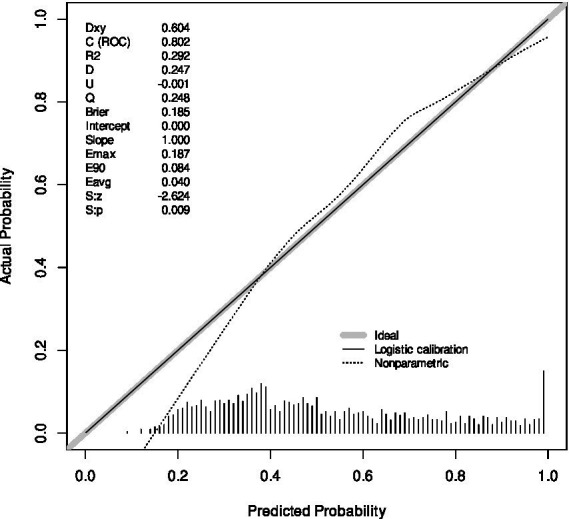
The calibration curve of the training set has a *p*-value greater than 0.05 in the Hosmer Lemeshow test.

**Figure 8:**
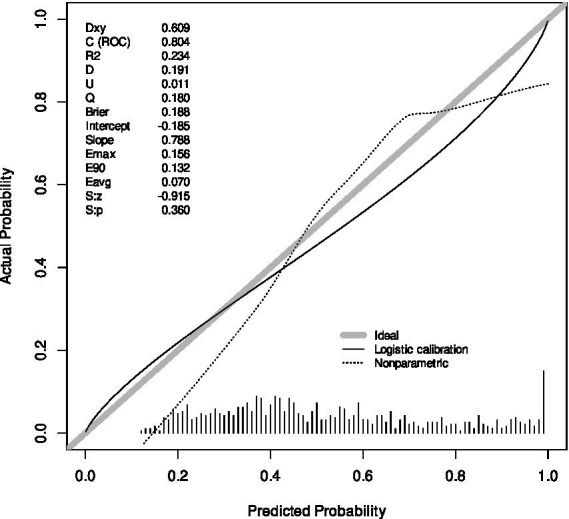
Calibration curve of the nomogram in the validation set. The diagonal dashed line (“ideal”) represents perfect calibration (predicted probability equals observed probability). The green line (“apparent”) shows the unadjusted calibration performance, while the blue line (“bias-corrected”) applies bootstrap correction (1,000 resamples) for optimism. The closer the bias-corrected curve lies to the ideal line, the better the calibration.

Analysis of the calibration curves and related metrics (Figures 7, 8) shows that neither the calibration curve for the training set nor that for the internal validation set perfectly aligns with the ideal 45° line. The Brier index for the training set is 0.185, and that for the validation set is 0.188. The slope of the calibration curve for the training set deviates significantly, while the calibration curve for the validation set fits better.

### 3.7 Decision curve analysis (DCA) of predictive models

The DCA curve results for the training set (Figure 4) and the internal validation set (Figure 5) show that, across a wide range of high-risk thresholds and cost-effectiveness ratios, the net benefit of the combined predictive model is significantly higher than that of the two extreme strategies: “treating all patients” and “treating no patients.” This indicates that, in clinical practice, applying this model for cross-sectional risk stratification in Parkinson’s disease can yield a positive net benefit for clinical decision-making and holds high clinical value.

## 4 Discussion

This study successfully developed and internally validated a parsimonious, clinically accessible nomogram for estimating an individual’s probability of currently having Parkinson’s disease (cross-sectional discrimination). By employing LASSO regression for variable selection from a comprehensive set of clinical and laboratory parameters, we identified and subsequently confirmed three independent core associated factors: a history of smoking, elevated Systemic Inflammation Index, and a lower Prognostic Nutritional Index. The composite model integrating these factors demonstrated moderate discriminatory performance, with an area under the curve of approximately 0.80 in both the derivation and validation cohorts. While SII_100_ exhibited the strongest individual predictive capacity, the integrated nomogram, as evidenced by decision curve analysis, provided superior clinical utility across a realistic range of threshold probabilities, offering a practical tool for individualized cross-sectional diagnostic classification.

The inverse association between smoking history and PD risk observed in our model robustly aligns with one of the most consistent epidemiological findings in neurology. This protective link is supported by large cohort studies and neuroimaging evidence indicating that smokers may have relatively preserved striatal dopamine transporter binding compared to non-smokers (Wang et al., 2022). The precise mechanism remains debated, but it is increasingly clear that nicotine alone is unlikely to be the primary mediator, given the negative results from clinical trials like NIC-PD (Rose et al., 2024a). Our finding reinforces the hypothesis that other components of tobacco smoke, or behaviors correlated with smoking, confer this epidemiological protection.

Concurrently, our results strongly implicate systemic inflammation as a key risk factor, quantified here by the SII_100_. Elevated SII_100_ was the most powerful single predictor in our analysis. This aligns with a substantial body of evidence characterizing PD as a condition involving both central and peripheral immune dysfunction (Tansey et al., 2022). Meta-analyses confirm altered levels of inflammatory cytokines such as IL-6 and TNF-*α* in the blood and cerebrospinal fluid of PD patients (Qu et al., 2023). The SII_100_, integrating neutrophils, platelets, and lymphocytes, may offer a more holistic snapshot of the systemic inflammatory milieu than individual markers. It is noteworthy that while some studies, including a large analysis from the United Kingdom Biobank, reported an inverse association between C-reactive protein and future PD risk (Li et al., 2024), our use of a cellular composite index (SII_100_) may capture a distinct aspect of immune activation related to PD pathogenesis.

The third pillar of our nomogram, a lower PNI, underscores the critical intersection of nutritional status and inflammation in PD susceptibility. PNI, derived from albumin and lymphocyte counts, reflects both nutritional depletion and a pro-inflammatory state. Our finding that poorer nutritional-inflammation status is independently associated with higher PD risk corroborates recent research highlighting the prognostic value of various inflammation-nutrition-based indicators, including PNI, for mortality in PD patients (Jia et al., 2024). Furthermore, pro-inflammatory dietary patterns, as measured by the Dietary Inflammatory Index, have been linked to a higher probability of prodromal PD (Balomenos et al., 2022). This suggests that chronic, subclinical malnutrition coupled with inflammation may create a permissive environment for neurodegenerative processes.

The biological pathways linking these three associated factors are likely interconnected and central to current models of PD etiology. The protective effect of smoking may be mediated not by nicotine but by other constituents, such as low-dose carbon monoxide, which in rodent models activates heme oxygenase-1 signaling, reduces *α*-synuclein pathology, and confers neuroprotection—effects commensurate with the reduced PD risk in smokers (Rose et al., 2024b). Systemic inflammation, indicated by a high SII_100_, can fuel neuroinflammation by compromising the blood–brain barrier, facilitating the entry of peripheral immune cells or inflammatory mediators into the central nervous system, thereby activating microglia and astrocytes in a chronic, deleterious manner (Tansey et al., 2022; Kip and Parr-Brownlie, 2022). This neuroinflammatory environment is known to exacerbate α-synuclein aggregation and neuronal vulnerability.

Nutritional status acts at this intersection. Poor nutrition, reflected by a low PNI, can impair intestinal barrier function, potentially contributing to gut dysbiosis and increased permeability. This may allow pro-inflammatory bacterial products to enter systemic circulation or activate the enteric nervous system, fueling the gut-brain axis of inflammation—a pathway strongly implicated in PD (Wang et al., 2021; Tan et al., 2022). Alterations in gut microbiota composition, including reduced levels of short-chain fatty acid producers like *Faecalibacterium*, have been consistently associated with PD and may link dietary patterns, inflammation, and neurodegeneration (Aho et al., 2021; Kleine Bardenhorst et al., 2023). Thus, the triad identified—smoking (protective), inflammation (detrimental), and nutrition (protective when adequate)—likely converges on modulating neuroinflammatory tone and protein homeostasis.

From a clinical perspective, the major strength of this nomogram lies in its simplicity and the routine availability of its components. Smoking history is a standard part of medical history, while SII_100_ and PNI are calculated from ubiquitous complete blood count and albumin measurements. This makes the tool highly accessible and cost-effective for potential use in primary care or community health screening settings. The ability to generate a personalized risk score can help identify individuals with a higher probability of a current PD diagnosis, who might be prioritized for neurological referral. The model does not predict future disease onset, which would require prospective cohort studies. The model tangibly highlights modifiable factors. It reinforces the potential importance of addressing systemic inflammation and ensuring optimal nutritional status, not merely for general health but as plausible strategies for PD risk reduction, aligning with calls for lifestyle interventions targeting neuroinflammation (Kip and Parr-Brownlie, 2022; Veronese et al., 2024). A critical consideration for real-world implementation is the selection of an appropriate risk probability threshold. The nomogram does not impose a single “optimal” cutoff; rather, the threshold should be chosen based on the clinical context. As shown in Table 4, a low threshold (≈30%) achieves high sensitivity (94.8%) but low specificity (33.0%). This setting is analogous to a screening test in primary care, where the priority is to avoid missing potential PD cases, and false positives are acceptable because they can be triaged to a specialist for further evaluation. Conversely, a higher threshold (≈60%) offers high specificity (87.7%) at the expense of sensitivity (53.3%). This would be appropriate in a specialist clinic where a positive nomogram result could prompt early advanced imaging or biomarker testing, while a negative result would argue against a PD diagnosis. The decision curve analysis (Figures 4, 5) confirms that the nomogram provides positive net benefit across a broad range of thresholds, without mandating a single cutoff. Clinicians and health systems are encouraged to calibrate the threshold according to local disease prevalence, the availability of confirmatory tests, and the consequences of false decisions. Future implementation studies should prospectively evaluate different thresholds in real-world primary and secondary care settings.

Several limitations of our study must be acknowledged to contextualize the findings. First, the retrospective, cross-sectional design at diagnosis limits causal inference. While we interpret SII_100_ and PNI as potential risk factors, they could equally represent consequences of early, subclinical disease processes or prodromal non-motor symptoms affecting nutrition and immune function. Second, the model was developed and validated within a single cohort; external validation in independent, ethnically diverse populations is essential to confirm generalizability. Third, while we controlled for several key confounders, residual confounding from unmeasured variables such as detailed dietary habits, specific environmental toxin exposures, or genetic predisposition cannot be excluded. For instance, genetic variation in loci like *HLA-DRB1* is known to interact with smoking in modifying PD risk (Domenighetti et al., 2022), and such interactions were not assessed here. Fourth our model was developed and internally validated using a single-center cohort from Chengdu Seventh People’s Hospital. The “internal validation” performed by random split of the same dataset does not substitute for external validation. Therefore, our findings should be considered hypothesis-generating, and external validation in independent, multicenter, and ethnically diverse populations is mandatory before any clinical application. Importantly, because of the cross-sectional design, our nomogram should be interpreted as a diagnostic discrimination tool (estimating the probability of already having PD) rather than a true risk prediction model for future disease onset.

Future research directions are clearly delineated by these findings and limitations. Prospective cohort studies with repeated biomarker measurements are needed to establish the temporal relationship between rising SII_100_, declining PNI, and the subsequent onset of clinical PD. Integrating genetic data, particularly polygenic risk scores or specific variants in immune-related genes like *LRRK2* (Herrick and Tansey, 2021; Tsafaras and Baekelandt, 2022), could create more powerful, integrative prediction models and illuminate critical gene–environment interactions. Exploring the dynamic changes of these indices across the prodromal and early clinical stages of PD, and their correlation with motor and non-motor symptom progression, would be highly informative (Kim et al., 2022; Kouli et al., 2024). Ultimately, the clinical utility of this nomogram should be tested in prospective primary care settings, and interventional studies should investigate whether modulating systemic inflammation or improving nutritional status in at-risk individuals can truly modify the trajectory toward PD. This clinically accessible nomogram may facilitate cross-sectional diagnostic discrimination for Parkinson’s disease in primary care or community-based screening settings. Prospective external validation is required before it can be considered for any future risk prediction purpose.

## Data Availability

The original contributions presented in the study are included in the article/supplementary material, further inquiries can be directed to the corresponding author.
